# Antidotal Method*Read before the Brooklyn Hahnemannian Union.

**Published:** 1898-03

**Authors:** B. Fincke

**Affiliations:** Brooklyn, N. Y.


### ANTIDOTAL METHOD.*

B. Fincke, M. D., Brooklyn, N. Y.

A case of hemicrania mercurialis cured with two pellets of Mercurius-vivus, 3,000 (F.), in 1856, in the Brooklyn Homœopathic Dispensary, has been recorded in *High Potencies and Homœopathics,* 1865, p. 8.

A case of Rhus poisoning was rapidly cured later with Rhus-tox., Lippe's Distillate 97m (F.), by Dr. Lippe. In 1865 poisoned again. Rhus-tox., 103m (F.) and 60m (F.) did not help any more, and Dr. Raue cured it with Anacardium-orientale.

A case of Rhus poisoning from collecting the poison-oak in the New Jersey swamps in 1865, causing itching eruption of blisters all over the hands in and outside, beginning at the wrists, which, after breaking, produced a bad odor and formed thick scabs, after using all and everything externally and internally for two weeks, yielded rapidly to a dose of Rhus-toxic. 60m (F.). Being deprived of sleep for two weeks, patient slept well the first night. After a second dose, 40m (F.), patient was healed in six days. *Medical Investigator,* Vol. VI, p. 6.

These are some prior claims to antidotal treatment than those of Dr. Swan and Dr. Sawyer, though they never were made, and considered only as simple homœopathic treatment.

To this comes what Bœnninghausen says in his *Aphorisms of Hippocrates:*"

"The antidotal power of a substance depends entirely upon its characteristic property of acting upon the living organism, of course after the eventual chemical or mechanical action is removed and only the dynamical remains. For this reason there are not only no universal antidotes against all

*Read before the Brooklyn Hahnemannian Union.

poisons, just as there is not or never can be any universal medicine against all diseases, but even for the various poisoning symptoms of one and the same medicine different antidotal poisons are necessary, of which every one again has its particular and exclusive range of action, beyond the limits of which its power does not extend." (Book V, p. 271.)

Speaking of the abuse of Iodine in diseases of enlargement and induration, he remarks that the worst of its action is the uncommon intensity and obstinacy, so that it is extremely difficult to find antidotes for it. "We have," he continues, "in such cases besides Hepar and Arsenicum, the best result from the highest potencies of the same remedy in repeated but smallest doses, dissolved in water, with the precaution, before taking the medicine, to shake the vial containing the fluid several times in order to exalt the dynamization somewhat, because otherwise, according to experience, the continued use is not well borne." (Book VI, p. 416.)

To this passage Bœnninghausen makes the following note: "If there is any experience to prove not so much the more penetrating but rather the more extended efficacy of the highly potentiated medicine, this is especially the *antidotal power* which such remedies have gained against the ill effects of a former abuse of the *same* medicine. In such cases the repeated administration of the same remedy in the *lowest attenuations* causes each time a distinct aggravation without following improvement; whereas, if given in *high potency*, though not always, yet for the most part it produces a more agreeable and extensive improvement than most other antidotes which meet and annihilate only those complaints within their range, but remain without effect beyond it."

Bœnninghausen's work was printed in 1863, and it is therefore probable that he has practiced what is now claimed as a distinct branch of Homœopathy under the name of antidotal method a long time before, because it is to be expected from a practitioner of well-known sagacity that he would not have spoken of this topic in such a comprehensive manner if he had not a trusty experience behind him.

Now, since questions of priority generally do not amount to much, and it is difficult to settle the merit of the invention upon an individual because there may be no record of it, it would be well to let it rest upon our good old friend Bœnninghausen, and with it lay the ghost which can only lead to useless controversy and leave the value of the invention itself out of question.

That it is a question of importance is true, and its discussion will no doubt lead in time to a proper application in practice if its limits are clearly defined.

There is no doubt, as Hahnemann has already demonstrated, that the simillimum is the true antidote, and if so, it will also be found the best prophylactic.

